# Supplementary material for: Lysis Cassette-Mediated Exoprotein Release in Yersinia entomophaga Is Controlled by a PhoB-Like Regulator
Source: Microbiol Spectr. 2023 Mar 23;11(2):e00364-23. doi: 10.1128/spectrum.00364-23 (PMC10101115; doi:10.1128/spectrum.00364-23)
Supplement: Supplemental file 2 — Fig. S1 to S7 and Tables S1 to S5. Download spectrum.00364-23-s0002.pdf, PDF file, 1.6 MB [file spectrum.00364-23-s0002.pdf]

Supplementary material

Lysis cassette-mediated exoprotein release in *Yersinia entomophaga* is controlled by a PhoB-like regulator

Marion Schoof[a,#,] Maureen O'Callaghan[a], Charles Hefer[a], Travis R. Glare[b], Amber R. Paulson[c], and Mark R.H. Hurst[a#]

[a] AgResearch, Resilient Agriculture, Lincoln Research Centre, Lincoln, New Zealand

[b] Wine and Molecular Biosciences Dept, Faculty of Agriculture and Life Sciences, Lincoln University, Lincoln, New Zealand

[c] British Columbia Ministry of Environment and Climate Change Strategy, Victoria, BC, Canada

Short title: *Regulation of* exoprotein release in *Y. entomophaga*

[#]Corresponding author:

Marion Schoof, AgResearch, Private Bag 4749, Christchurch 8140, New Zealand, +64 3 321 8740, marion.schoof@agresearch.co.nz

Mark RH Hurst, AgResearch, Private Bag 4749, Christchurch 8140, New Zealand, +64 3 325 9919, mark.hurst@agresearch.co.nz

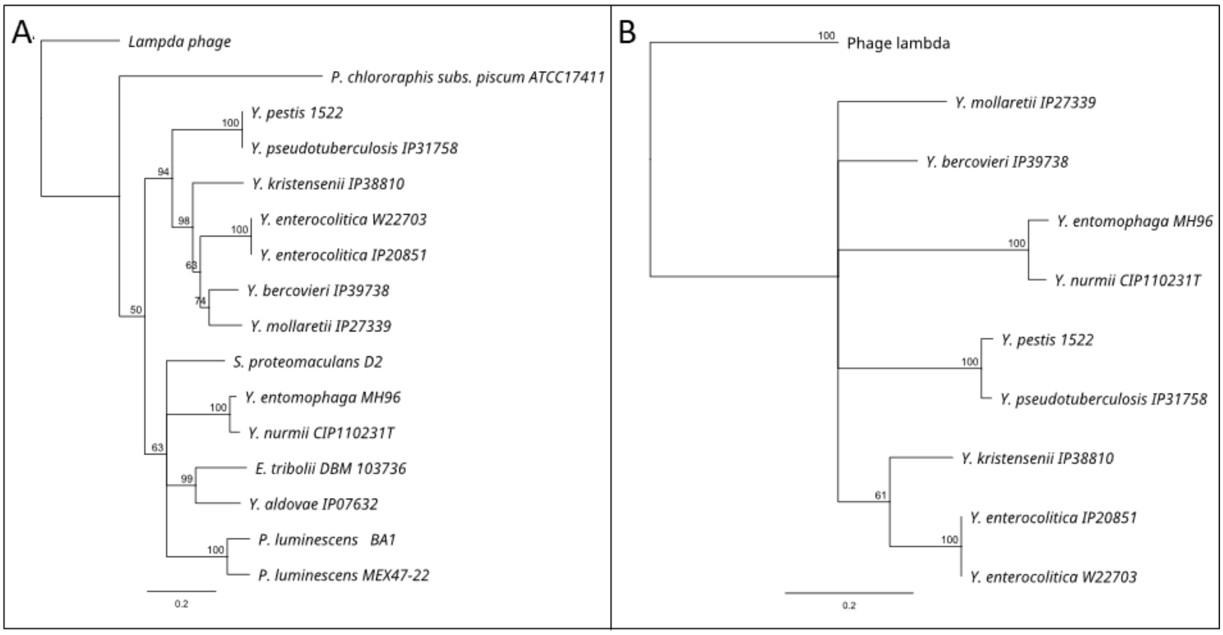

Figure S1: Phylogeny of nucleotide comparison of ALC genes encoding HolA (A) and PepB (B); maximum likelihood, 1000 bootstrap replicates, bootstrap values indicated at respective nodes, scale bar denoting patristic distances.

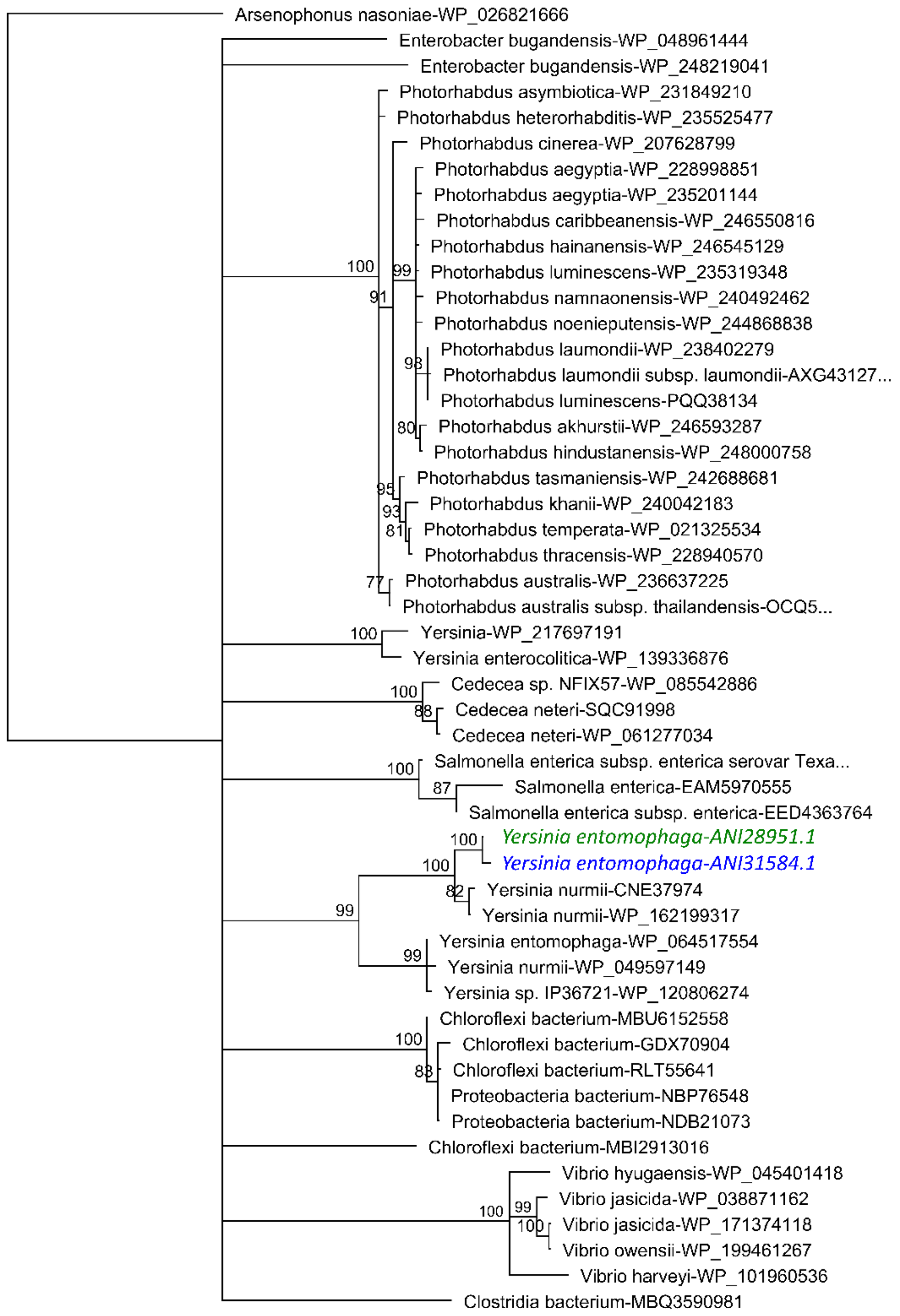

Figure S2: Phylogenetic analysis of RoeA orthologs. Amino acid sequences were aligned using MUSCLE and a phylogenetic tree was inferred using the neighbour-joining method, with 1000 bootstrap replicates indicated at respective nodes. Closest *Y. entomophaga* RoeA (blue) ortholog is *Y. entomophaga* Yen7 (green), which form a clade with *Y. nurmii*. Scale bar denoting patristic distances.

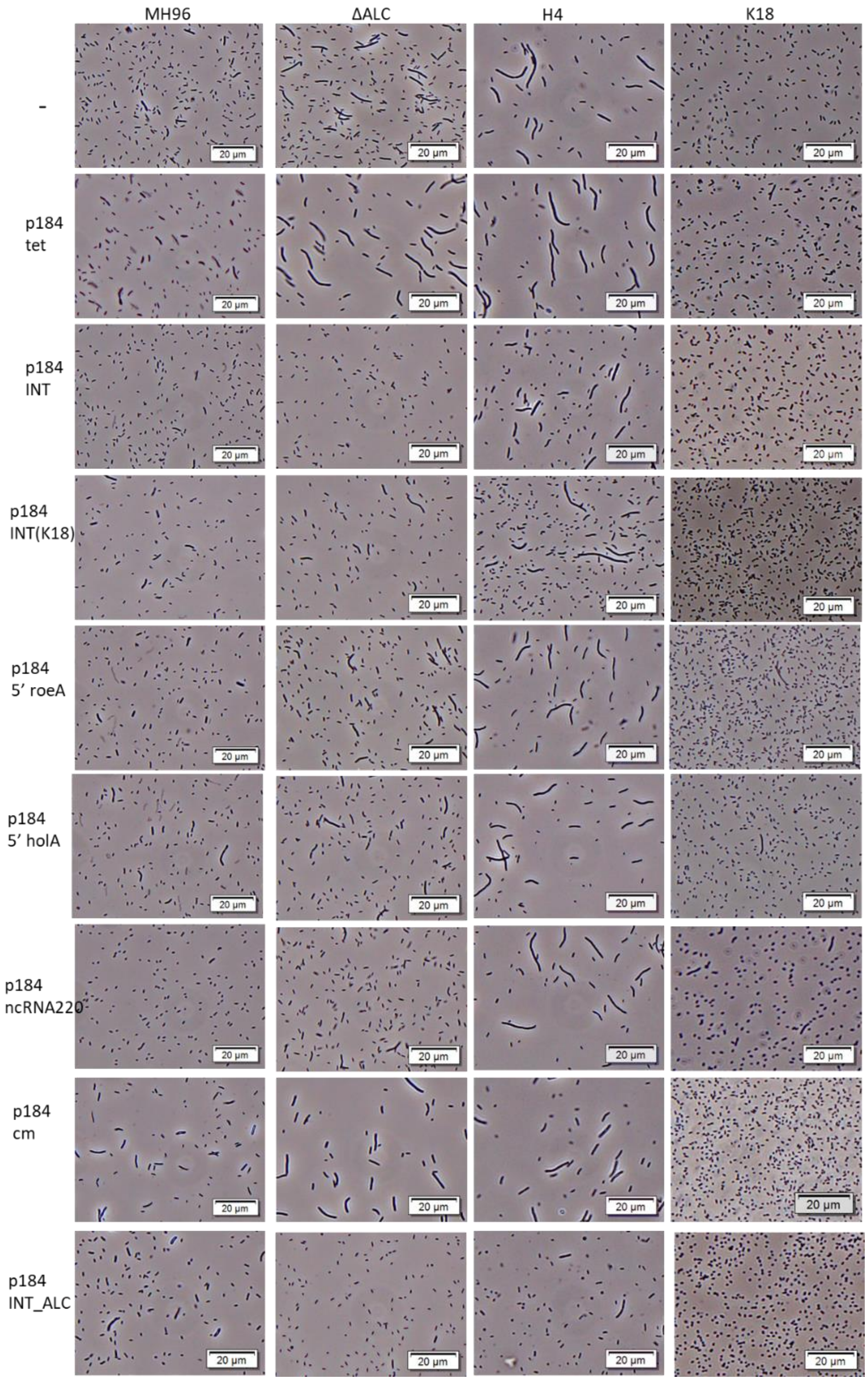

Figure S3: Light microscopy of in *trans* complementation with the listed p184 vector series (p184INT, p184 INT(K18), p184 5’*roeA,* p1845’*holA,* p184ncALC220, p184ncALC673, p184INT_ALC) used for *trans* complementation of the YeRER and the pACYC184 (p184)control vector grown in the presence of 10 µg/mL tetracycline (tet) or 30 µg/mL chloramphenicol (cm) in either MH96, K18, H4 (5’ UTR *holA* mutant) and ΔALC. MH96, K18, H4 and ΔALC without vector are shown and indicated by (-). Scale bar: 20 µm.

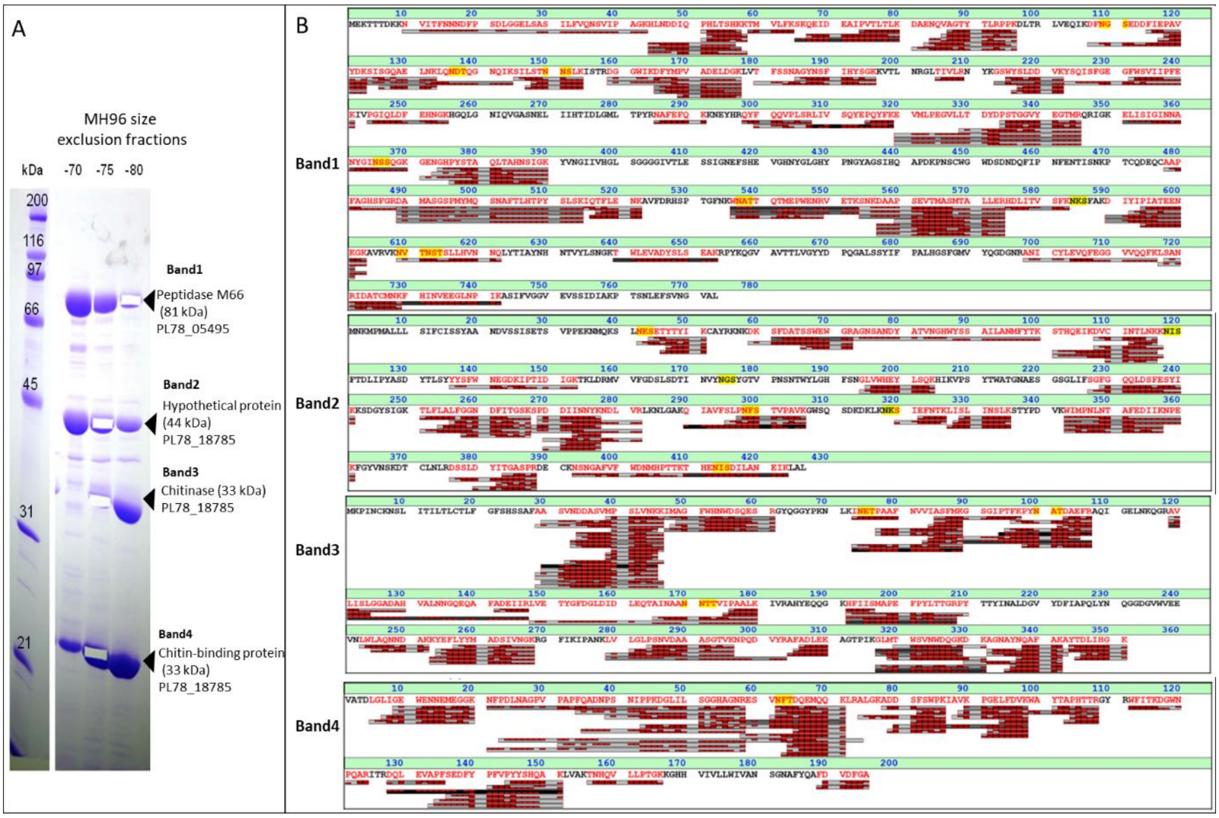

Figure S4: Characterization of MH96 prominent exoproteins. A) Size-exclusion chromatography fragment pools from cell supernatant of *Y. entomophaga* MH96 visualized on 12 % polyacrylamide gel by SDS-PAGE and stained with Coomassie brilliant blue. Excised bands used for LC-ESI-MS/MS are indicated by black arrows and the resultant LC-ESI-MS/MS data listed. B) LC-ESI-MS/MS peptide alignment with bands excised from gel in Fig. S4. Band 1: Peptidase M66 (PL78_0545), Band 2: peptide alignment with Hypothetical protein (PL78_18785), Band 3: peptide alignment with Chitinase (PL78_11910), Band 4: peptide alignment with Chitin binding protein (PL78_05310).

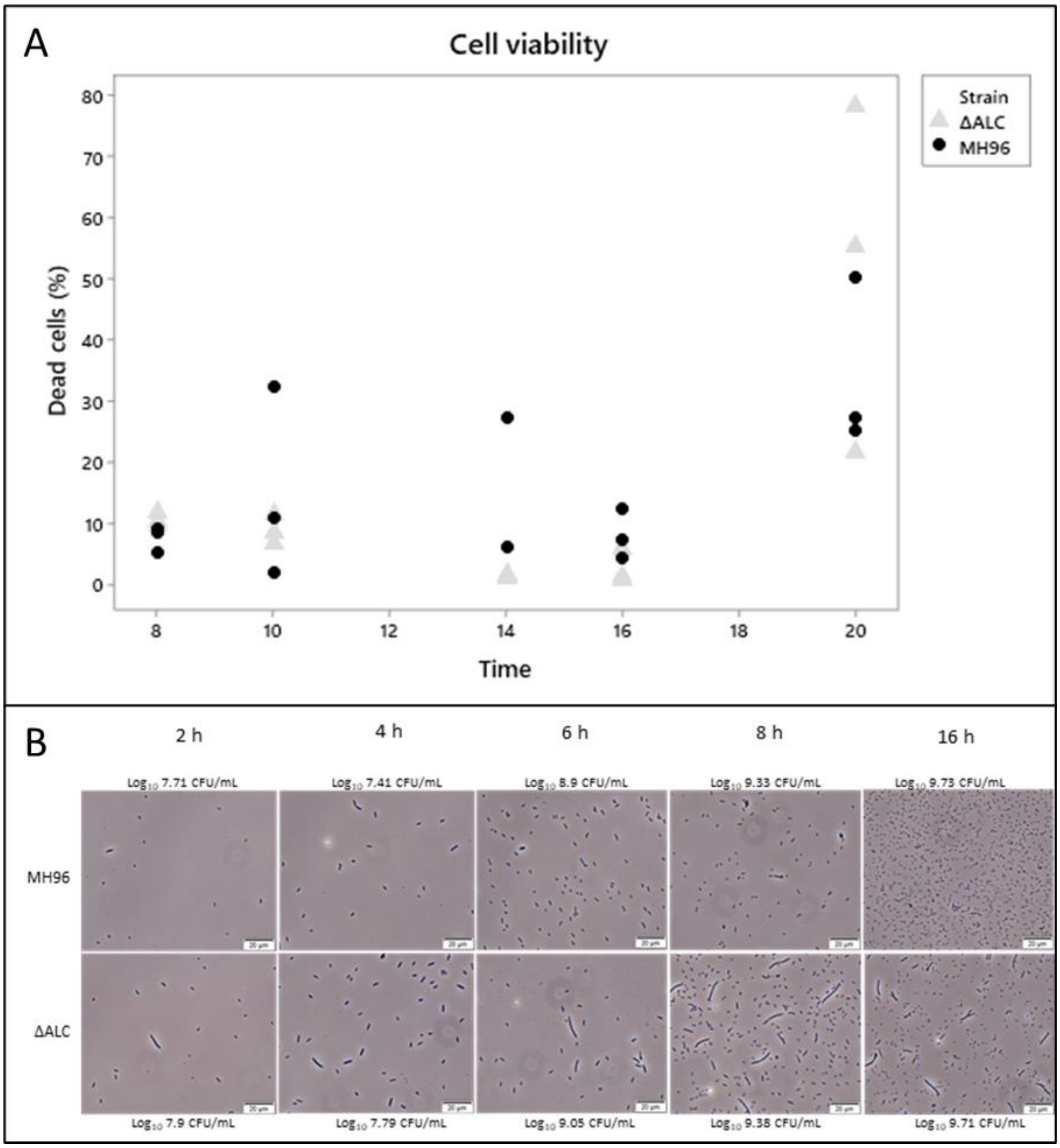

Figure S5: Cell viability and growth assessment in MH96 and ΔALC. A) Cell viability assessed using the LIVE/DEAD stain from LB broth cultures taking at the same time points as MH96. Cell viability is presented as proportion to the total cell count measured in 9 independent samples per strain at each time point. B) Light microscopy of time-dependent cell morphology, CFU of MH96 and ΔALC are provided. The increasing proportion of elongated ΔALC cells can be observed through time. Scale bar: 20 µm.

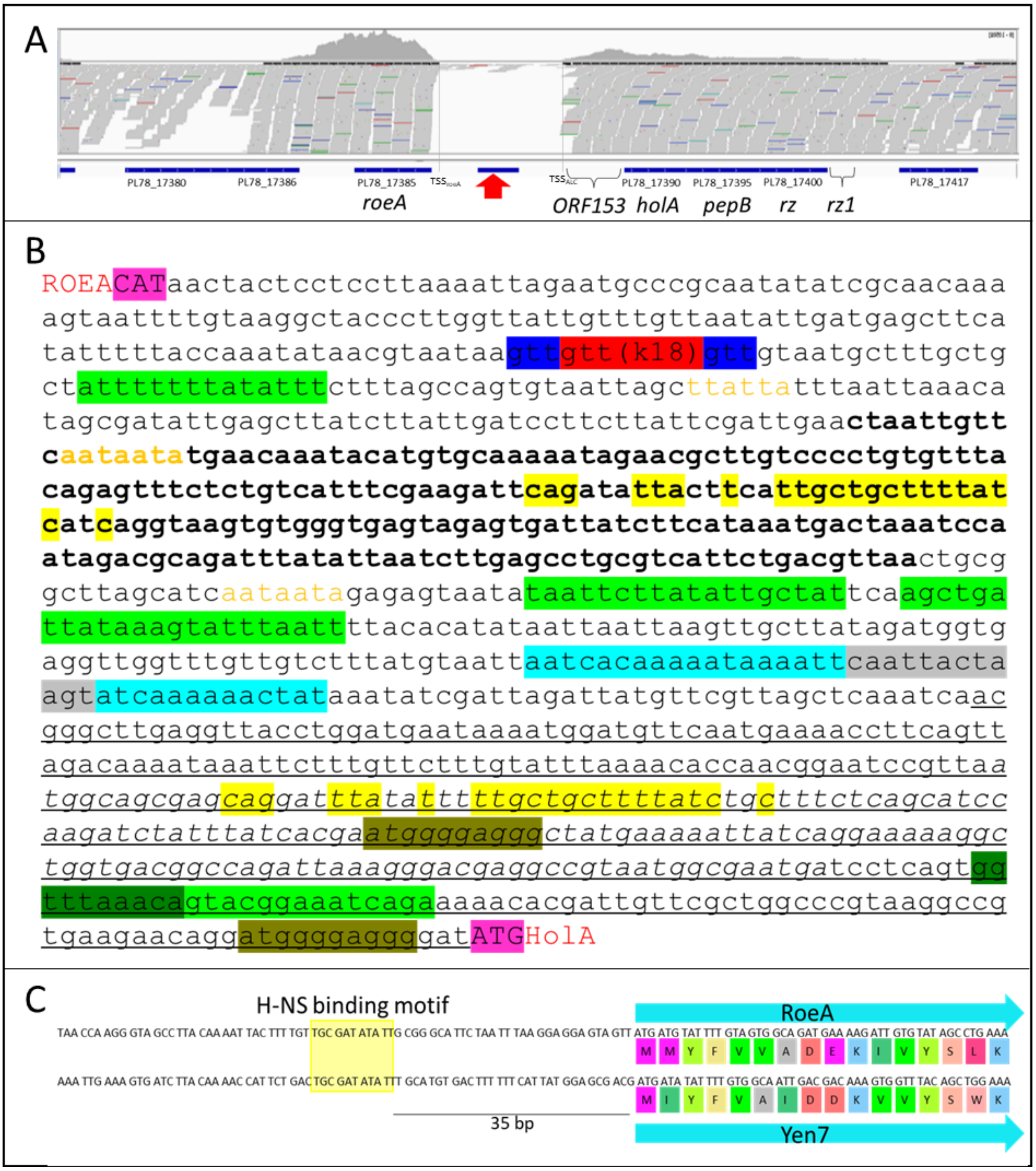

Figure S6: Nucleotide analysis of YeRER intergenic region (INT). A) Read mapping of *in vitro* transcriptome analysis of the YeRER in MH96. Vertical red arrow denotes location of the predicted ncRNA (ncALC220); Location of predicted ORF153. B) Adjacent gene *holA* and *roeA* indicated (red letters). Hfq binding motifs based on Lorenz et al. (1) in orange letters. 5'UTR of *holA* indicated (underlined). Highlighted are the following: base pair deleted in K18 (red) within a 3 bp repeat region (dark blue); degenerate long repeat (yellow); hairpin structure (light blue) with loop (gray); PhoB and PhoB-like DNA binding motif (light green) – overlapping PhoB-binding motifs were identified within the hairpin structure and for visualization purpose not highlighted here; H-NS binding motif (dark green); perfect repeats (kaki). C) Homologous H-NS binding sequence 5' *roeA* and *yen7* based on Lang et al. (2).

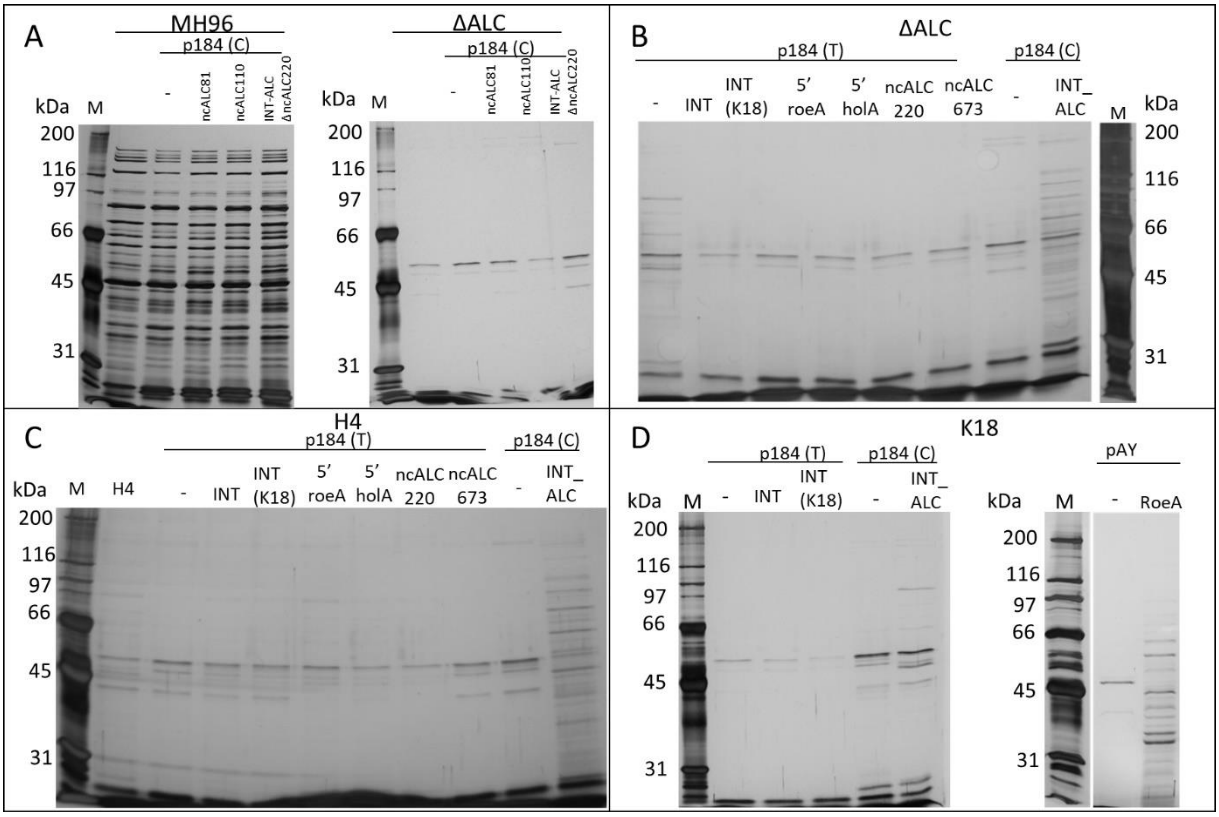

Figure S7: 10% SDS-PAGE of the *trans* complementation of p184 constructs in MH96 (A), ΔALC(A,B), H4 (C) and K18 (D). Samples are taken from supernatant of late exponential phase cell cultures. Cells were grown with chloramphenicol 30 µg/ml (C) or tetracycline 10 µg/mL (T), except for wild type strains MH96, and ΔALC and H4 which were grown without antibiotics. K18 cells grown with pAY vector in (D) were grown with ampicillin 100 µg/mL.

Table S 1: Bacteria strains used in this study

Strains	Description	Reference
E. coli		
ST18	*Escherichia coli* S17 λpir Δ*hemA*	(3)
DH10β	F- *mcrA* Δ(*mrr-hsdRMS-mcrBC*) Φ80d*lacZ*ΔM15 Δ*lacX*74 *endA1 recA1 deoR* Δ(*ara,leu*)7697 *araD*139 *galU galK nupG rpsL* λ-	(4)
CM2929	Cm^{R}, *dam*13::Tn*9*, *dam*-6	(5)
Y. entomophaga		
MH96	Wild-type strain, isolated from diseased *Costelytra giveni* larva	(6)
ΔALC	Mh96 mutation by deletion of *holA,pepB,rz* and included deletion of partial *rz1* including rbs and initiation codon.	This study
K18	Spontaneous MH96 non-secreting derivative, isolated at 4 weeks post field trial application.	AgResearch culture collection
H4	Transposon mutant of MH96 generated by Tn5 insertion in 120 bp 5' *holA*	(7)
H12	Transposon mutant of MH96 generated by Tn5 insertion at 138 bp within *roeA*	(7)
MH96Δ*roeA151::Spec*	Sp, *roeA* mutation by gene disruption using sp-cassette insertion at position 151 nt of *roeA*	This study

Table S2: Plasmids used throughout this study

Plasmid	Description	Source or reference
pKD4	Kan, cloning vector	(8)
pGEM T-Easy	Amp, cloning vector, LacZ multi-cloning site	Promega Ltd.
pJP5608	Tet, suicide vector,	(9)
pAY2-4	Amp, arabinose induction vector	(10)
pACYC184	Cm, Tet, cloning vector	(11)
pHP45	Spec, cloning vector	(12)
pBAD::sfGFP	Amp, sfGFP cloning vector	(13)
pGEMΔALC	Kan, Amp, cloning vector of deletion *holA/pepB/rz* by replacing with kanamycin cassette; ALC flanking regions 1.5 kb (ΔALC amplicon)	This study
pJP5608ΔALC	Tet, Kan, suicide vector, ΔALC amplicon	This study
pAY-ALC-opt	pAY2-4, expression vector of optimised ALC coding region expressing *holA/pepB/rz/rz1*	This study
pAY-ALCΔ*rz1*	pAY2-4, expression vector of *holA/pepB/rz*	This study
pAY- ALCΔ*rz1-opt*	pAY2-4 expression vector of optimsed ALC expressing *holA/pepB/rz*	This study
pAY- ALCΔ*rz*-opt	pAY2-4 expression vector of optimsed ALC expressing *holA/pepB/rz1*	This study
pAY- ALCΔ*pepB*-opt	pAY2-4, expression vector of optimsed ALC expressing *holA/rz/rz1*	This study
pAY-RoeA	pAY2-4, expression vector of RoeA	This study
P184-INT	pACYC184, tet, expressing YeRER intergenic region from MH96 (1038 bp)	This study
P184-INT(K18)	pACYC184, tet, expressing YeRER intergenic region from K18 (1035 bp)	This study
P184-INT_ALC	pACYC184, cm, expressing YeRER intergenic region and *holA/pepB/rz* from MH96 (2133 bp)	This study
P184-5'holA	pACYC184, tet, expressing YeRER intergenic region of 5' *holA* from MH96 (571 bp)	This study
P184-5'roeA	pACYC184, tet, expressing YeRER intergenic region of 5' *roeA* from MH96 (467 bp)	This study
P184-ncALC220	pACYC184, tet, expressing ncRNA from YeRER intergenic region (220 bp)	This study
P184-ncALC81	pACYC184, tet, expressing truncated ncRNA from YeRER intergenic region (81 bp)	This study
P184-ncALC673	pACYC184, tet, expressing partial YeRER intergenic region including ncRNA220 from MH96 (673 bp)	This study

Table S3: GeneArt (GA) (Thermo Fisher Scientific, USA) and GenScript (GS) (USA) synthesized ALC constructs which were cloned into pAY. Underline indicates restriction sites, italics indicate ribosomal binding site and bold indicates start codon. Blue letters indicate HolA encoding region, green letters indicate PepB encoding region, red letters indicate Rz coding region and pink letters indicate Rz1 encoding region.

ALC -opt<br><br>(GA)	CAT**ATG**ACGTTAAAGATTCCGACTGATACACAGGCAATTACCTGGTTAATTATTGGCCTTTTTTCAG<br>CTTGGGGAGGTGTTGTGAGATATCTCATGGATATGCAAGGGCACAGGGGGAGTTGGAGTTGGAT<br>GGGAATAATAAGTCAGATCATTATTTCAAGCTTCACTGGCCTTTTGGGGGGATTACTCAGCTTTGA<br>AAATGGCAGTAGTCATTACATGACTTTTGCTATTGCAGGCTTATTTGGCACCTTGGGGAGTACGGC<br>GTTGAGTTACCTGTGGCGGCGTTTCTTGGGGAGTCCCGAAAAGAACCGTGGGTAAAG*GAGGA*AA<br>AAC**GTG**GGTAACTTTACTTTTAGTCAAATCAGTCAACGCAATCTACAAGGCATACACCCGGATCTC<br>GTTCGGGTGGTGTACCTGGCACTCAAATTGTCCGAGGTGGATTTTCGCGTTATCGAAGGGCTGCG<br>TGATAGCGCACGTCAACGGCAAATGGTGCTTAATGGCAAGAGCCAAACGCTCAATAGCCGGCATC<br>TTACCGGTCATGCGGTCGATTTGGCGCCTTGGTTCAACAACACGATTCCCTGGAACGATTGGGGA<br>GCGTTTGCTCAGGTTGCAGCGGCGATGAAACAGGCGGCGAAGCAACTACAAATTCCTGTGATCTG<br>GGGCGGTGACTGGACGACGCTTAAGGATGGGCCGCACTTTGAGTTGCCCCGTATGCAGTATCCTT<br>GAA*GGAGGA*AAAAA**ATG**AGTCTGCTCAGTGTTTTGAAACGTGGTCTGCTACTTGCCACGCTATTT<br>GCGGTTTTGGTTGGCGGGCTGTGGGTTGCTCAACTGAAAAAGACCGCGACATTACTGAATTCTGA<br>AAATATCAGTCTGACGCAGCAGAATCAGCTTTATCTGCAACGGTTGCAAGGATATGACCAGCAGA<br>TGAAAACACTGGATGAGGCGTTAACCACTAATGCCATAACTCAGCGTCAGGCAGAGGAGAAATAT<br>CATGCGATCGAACAACAAATGCGGCGGCTGCTGGCTAATAGCCGCTGCGCTCGGGAGCCTGTGC<br>CTGATGCTGTTATCCGGTTGCAGCAACGAGCACTTGGCCAAAGCCTGCCCGGTGCAGCCAATAGT<br>ACCGAAAGCGCTGCTATTGCCCCATGACA*GAGGA*GAAATATC**ATG**CGATCGAACAACAAATGCG<br>GCGGCTGCTGGCTAATAGCCGCTGCGCTCGGGAGCCTGTGCCTGATGCTGTTATCCGGTTGCAGC<br>AACGAGCACTTGGCCAAAGCCTGCCCGGTGCAGCCAATAGTACCGAAAGCGCTGCTATTGCCCCA<br>TGATCCCCCGTTATTTAACGTCACAAAGTGGGGGGACTATCCCGGTTATGTGGAAGAATTAAATCT<br>ATCGTTGGCGCGTTGTAATGCAGATAAAACGGCGGTGGCCCAAATAATGTCTCCCCAAAAAGGAT<br>AATCCCGGATCCCTCGAG
ALCΔ*pepB*-opt<br><br>(GS)	CAT**ATG**ACGTTAAAGATTCCGACTGATACACAGGCAATTACCTGGTTAATTATTGGCCTTTTTTCAG<br>CTTGGGGAGGTGTTGTGAGATATCTCATGGATATGCAAGGGCACAGGGGGAGTTGGAGTTGGAT<br>GGGAATAATAAGTCAGATCATTATTTCAAGCTTCACTGGCCTTTTGGGGGGATTACTCAGCTTTGA<br>AAATGGCAGTAGTCATTACATGACTTTTGCTATTGCAGGCTTATTTGGCACCTTGGGGAGTACGGC<br>GTTGAGTTACCTGTGGCGGCGTTTCTTGGGGAGTCCCGAAAAGAACCGTGGGTAAA*GGAGGA*AA<br>AAA**ATG**AGTCTGCTCAGTGTTTTGAAACGTGGTCTGCTACTTGCCACGCTATTTGCGGTTTTGGTT<br>GGCGGGCTGTGGGTTGCTCAACTGAAAAAGACCGCGACATTACTGAATTCTGAAAATATCAGTCT<br>GACGCAGCAGAATCAGCTTTATCTGCAACGGTTGCAAGGATATGACCAGCAGATGAAAACACTGG<br>ATGAGGCGTTAACCACTAATGCCATAACTCAGCGTCAGGCAGAGGAGAAATATCATGCGATCGAA<br>CAACAAATGCGGCGGCTGCTGGCTAATAGCCGCTGCGCTCGGGAGCCTGTGCCTGATGCTGTTAT<br>CCGGTTGCAGCAACGAGCACTTGGCCAAAGCCTGCCCGGTGCAGCCAATAGTACCGAAAGCGCT<br>GCTATTGCCCCATGACA*GAGGA*GAAATATC**ATG**CGATCGAACAACAAATGCGGCGGCTGCTGGCT<br>AATAGCCGCTGCGCTCGGGAGCCTGTGCCTGATGCTGTTATCCGGTTGCAGCAACGAGCACTTGG<br>CCAAAGCCTGCCCGGTGCAGCCAATAGTACCGAAAGCGCTGCTATTGCCCCATGATCCCCCGTTAT<br>TTAACGTCACAAAGTGGGGGGACTATCCCGGTTATGTGGAAGAATTAAATCTATCGTTGGCGCGT<br>TGTAATGCAGATAAAACGGCGGTGGCCCAAATAATGTCTCCCCAAAAAGGATAATCCCGGATCCC<br>TCGAG

ALCΔ*rz*-opt (GS)	CAT**ATG**ACGTTAAAGATTCCGACTGATACACAGGCAATTACCTGGTTAATTATTGGCCTTTTTTCAG<br>CTTGGGGAGGTGTTGTGAGATATCTCATGGATATGCAAGGGCACAGGGGGAGTTGGAGTTGGAT<br>GGGAATAATAAGTCAGATCATTATTTCAAGCTTCACTGGCCTTTTGGGGGGATTACTCAGCTTTGA<br>AAATGGCAGTAGTCATTACATGACTTTTGCTATTGCAGGCTTATTTGGCACCTTGGGGAGTACGGC<br>GTTGAGTTACCTGTGGCGGCGTTTCTTGGGGAGTCCCGAAAAGAACCGTGGGTAAAG*GAGGA*AA<br>AAC**GTG**GGTAACTTTACTTTTAGTCAAATCAGTCAACGCAATCTACAAGGCATACACCCGGATCTC<br>GTTCGGGTGGTGTACCTGGCACTCAAATTGTCCGAGGTGGATTTTCGCGTTATCGAAGGGCTGCG<br>TGATAGCGCACGTCAACGGCAAATGGTGCTTAATGGCAAGAGCCAAACGCTCAATAGCCGGCATC<br>TTACCGGTCATGCGGTCGATTTGGCGCCTTGGTTCAACAACACGATTCCCTGGAACGATTGGGGA<br>GCGTTTGCTCAGGTTGCAGCGGCGATGAAACAGGCGGCGAAGCAACTACAAATTCCTGTGATCTG<br>GGGCGGTGACTGGACGACGCTTAAGGATGGGCCGCACTTTGAGTTGCCCCGTATGCAGTATCCTT<br>GACA*GAGGAG*AAATATC**ATG**CGATCGAACAACAAATGCGGCGGCTGCTGGCTAATAGCCGCTGC<br>GCTCGGGAGCCTGTGCCTGATGCTGTTATCCGGTTGCAGCAACGAGCACTTGGCCAAAGCCTGCC<br>CGGTGCAGCCAATAGTACCGAAAGCGCTGCTATTGCCCCATGATCCCCCGTTATTTAACGTCACAA<br>AGTGGGGGGACTATCCCGGTTATGTGGAAGAATTAAATCTATCGTTGGCGCGTTGTAATGCAGAT<br>AAAACGGCGGTGGCCCAAATAATGTCTCCCCAAAAAGGATAATCCCGGATCCGGATCCCTCGAG
ALZΔ*rz1*-opt<br>(GA)	CAT**ATG**ACGTTAAAGATTCCGACTGATACACAGGCAATTACCTGGTTAATTATTGGCCTTTTTTCAG<br>CTTGGGGAGGTGTTGTGAGATATCTCATGGATATGCAAGGGCACAGGGGGAGTTGGAGTTGGAT<br>GGGAATAATAAGTCAGATCATTATTTCAAGCTTCACTGGCCTTTTGGGGGGATTACTCAGCTTTGA<br>AAATGGCAGTAGTCATTACATGACTTTTGCTATTGCAGGCTTATTTGGCACCTTGGGGAGTACGGC<br>GTTGAGTTACCTGTGGCGGCGTTTCTTGGGGAGTCCCGAAAAGAACCGTGGGTAAAG*GAGGA*AA<br>AAC**GTG**GGTAACTTTACTTTTAGTCAAATCAGTCAACGCAATCTACAAGGCATACACCCGGATCTC<br>GTTCGGGTGGTGTACCTGGCACTCAAATTGTCCGAGGTGGATTTTCGCGTTATCGAAGGGCTGCG<br>TGATAGCGCACGTCAACGGCAAATGGTGCTTAATGGCAAGAGCCAAACGCTCAATAGCCGGCATC<br>TTACCGGTCATGCGGTCGATTTGGCGCCTTGGTTCAACAACACGATTCCCTGGAACGATTGGGGA<br>GCGTTTGCTCAGGTTGCAGCGGCGATGAAACAGGCGGCGAAGCAACTACAAATTCCTGTGATCTG<br>GGGCGGTGACTGGACGACGCTTAAGGATGGGCCGCACTTTGAGTTGCCCCGTATGCAGTATCCTT<br>GAA*GGAGGA*AAAAA**ATG**AGTCTGCTCAGTGTTTTGAAACGTGGTCTGCTACTTGCCACGCTATTT<br>GCGGTTTTGGTTGGCGGGCTGTGGGTTGCTCAACTGAAAAAGACCGCGACATTACTGAATTCTGA<br>AAATATCAGTCTGACGCAGCAGAATCAGCTTTATCTGCAACGGTTGCAAGGATATGACCAGCAGA<br>TGAAAACACTGGATGAGGCGTTAACCACTAATGCCATAACTCAGCGTCAGGCAGAGGAGAAATAT<br>CATGCGATCGAACAACAAATGCGGCGGCTGCTGGCTAATAGCCGCTGCGCTCGGGAGCCTGTGC<br>CTGATGCTGTTATCCGGTTGCAGCAACGAGCACTTGGCCAAAGCCTGCCCGGTGCAGCCAATAGT<br>ACCGAAAGCGCTGCTATTGCCCCATGA

Table S4: Predicted protein binding sites located in the YeRER intergenic region of *roeA* and *holA* identified using CollecTF (14) and Prodoric programmes (15). Indicating binding sequence of known DNA-binding proteins in different gram-negative bacteria species, and location within the YeRER intergenic region.

Binding protein	Position within intergenic ReYER (nt)	Binding sequence	Source strain(s)
Binding sequences in intergenic region of YeRER			
PhoP	67 - 89<br>67 - 89<br>70-87	CCTTGGTTATTGTTTGTTAATAT<br>CCTTGGTTATTGTTTGTTAATAT<br>TGGTTATTGTTTGTTAAT	*E. coli K12*<br>*Salmonella …*<br>*Y. pestis.*
Fur	70-90<br>75-92	TGGTTATTGTTTGTTAAT<br>TGGTTATTGTTTGTTAATATT	*Dyckia*<br>*Y. enterocolitica*
OmpR	148-164<br>151-166	CTGCTATTTTTTTATAT<br>CTATTTTTTTATATTT	*Y. enterocolitica 8081*<br>*Y. enterocolitica YPIII*
RegA	153-170	CTATTTTTTTATATTT	*Citrobacter*
ArcA	155-175	ATTTTTTTATATTTCTTT	*E. coli K12*
RdgB	163-186	ATTTCTTTAGCCAGTGTAATTAGC	*Pectobacterium carotovorum subsp. carotovorum PCC21*
OxyR	155-188	TTTTTTATATTTCTTTAGCCAGTGTAATTAGCTT	*Pseudomonas putida KT2440*
Fur	485-505<br>488-518	AATAATAGAGAGTAATATAAT<br>AATAGAGAGTAATATAATTCTTATATTGCTA	*Dyckia*<br>*E. coli K12*
RegA	502-519	TAATTCTTATATTGCTAT	*Cirtrobacter*
PhoP	523-545<br>526-543	AGCTGATTATAAAGTATTTAATT<br>TGATTATAAAGTATTTAA	*E. coli K12*<br>*Y. pestis*
PhoB	523-545	AGCTGATTATAAAGTATTTAATT	*Salmonella*
ArcA	544-564	TTTTACACATATAATTAATTA	*E. coli K12*
CsgD	542-555	AATTTTACACATAT	*E. coli K12*
ArcA	591-611	TTGTTGTCTTTATGTAATTAA	*E. coli K12*
Fur	613-631<br>616-636<br>642-659	CACAAAAATAAAATTCAAT<br>AAAAATAAAATTCAATTACTA<br>CAAAAAACTATAAATATC	*Y. pestis*<br>*Dickya*<br>*salmonella*
ToxR	620-629	ATAAAATTCA	*V. cholerae*
PhoP	638-660<br>640-657<br>641-657	GTATCAAAAAACTATAAATATCG<br>ATCAAAAAACTATAAATA<br>TCAAAAAACTATAAATA	*Salmonella*<br>*Y. pestis*<br>*E. coli K12*
MntR	650-675	TATAAATATCGATTAGATTATGTTCG	*E. coli K12*
Fur	940-958	ATGGCGAATGATCCTCAGT	*Y. pestis*
ArcA	957-972	GTGGTTTAAACAGTAC	*V. fischeri*
HNS	959-968	GGTTTAAACA	*E. coli K12*
PhoP	960-982<br>960-982<br>962-980<br>962-980	GTTTAAACAGTACGGAAATCAGA<br>GTTTAAACAGTACGGAAATCAGA<br>TTAAACAGTACGGAAATCA<br>TTAAACAGTACGGAAATCA	*E. coli K12*<br>*Salmonella*<br>*Y. pestis*<br>*Y. pestis KIM*
Binding sequence 5′ yen7			
OmpR	66-79	TTTGAAATTGAA	*S. enterica*
	72-90	AATATAATTAGTTTGAAA	*S. enterica*
	103-121	GTTATTTTTTAATAAAAA	*E. coli*
	129-147	TAGAAATTATAATGTTAA	*E. coli*
PhoB	22-43	TTTTTAAATTAATGGTGGTATC	*E. coli*
	68-94	CTGGCTATAATGCTTAGCACTA	*E. coli*
	129-147	ATAGCTTAACTGAGAACCT	*E. coli*

106 Table S5: Primers used in this study

Primer	Sequence	Temp	Description	Note
MS01	GTGTAGGCTGGAGCTGCTTC	pKD4	fw primer FRT-Kan-FRT	FRT-site
MS02	CATATGAATATCCTCCTTAGTTCC	pKD4	rev primer FRT-Kan-FRT	
MS101	AAACATATGACGTTAAAGATTCCGACTG	MH96	fw pAY-holin(operon)	NdeI
MS104	AAACTCGAGGGATAATGCCGACACACTTTAA	MH96	rev pAY – hol+m15+lysB	XhoI
MS134	CTAATTGTTCAATAATATGAAC	MH96	ncRNA220 fw	
MS135	GTTAACGTCAGAATGACGCAG	MH96	ncRNA220 rev	
MS136	ATAGCGATATTGAGCTTATC	MH96	ncRNA81 fw 2	
MS137	CTTTATAATCAGCTTGAATAGC	MH96	ncRNA81 rev2	
MS138	GAGTTTCTCTGTCATTTCGAAG	MH96	ncRNA673 fw	
MS139	CGTGATAAATAGATCTTGGATG	MH96	ncRNA673 rev	
MS29	CCCTATCTATTAGCTGACCG	MH96	validation ΔALC fw	
MS30	CACGAACATGTAGAGCCAGC	MH96	validation ΔALC rev	
MS40	AAACCCGGGCGCGATTGTCTCCTCTTTTTG	MH96	1.949 bp 5’ *holA* fw	SmaI
MS41	AAACCCGGGCCCGATTAAGGAAACTTAG	MH96	2.039 bp 3’ *rz* rev	SmaI
MS42	AAACCCGGGCCCTCCCCATCCTGTTCTTC	MH96	2 bp’ 5 *holA* rev	SmaI
MS43	AAACCCGGGAACTACTCCTCCTTAAAATTAG	MH96	INT fw	SmaI
MS44	AAACCCGGGTCATGGGGCAATAGCAGCGC	MH96	*rz* rev	SmaI
MS45	AAACCCGGGATGATGTATTTTGTAGTGGCAG	MH96	*roeA* fw	SmaI
MS46	AAACCCGGGCCCCCGTTATTTAACGTCAC	MH96	3’ *rz* fw	SmaI
MS65	AAACAT**ATG**ATGTATTTTGTAGTGGCAG	MH96	RoeA for pAY fw	NdeI
MS66	AAACTCGAGTTAAAGTGTGTCGGCATTATC	MH96	RoeA for pAY rev	XhoI
MS71	CAATTACAACGTGGACCTCTGCCG	MH96	790 bp 3’ *roeA* rev	
MS72	TAAGCGTCGTCCAGTCACCGCCC	MH96	1.691 bp 5’ *roeA* fw	
MS74	AAAGTCGACCATAACTACTCCTCCTTAAAATT	MH96	lacZ-roeA fw	SalI
MS75	AAACCCGGGCCGCAGTTAACGTCAGAATG	MH96	lacZ-roeA rev	SmaI
MS82	GCGGCGGAGCTCCGGTAGTGGCATAGGGTT AG	MH96	2.308 bp 5’ *holA* fw	SacI
MS83	**GAAGCAGCTCCAGCCTACAC**CCCTCCCCATC CTGTTCTTC	MH96	5’ region *holA rev*	FRT
MS84	**ACTAAGGAGGATATTCATATG**CCCCCGTTATT TAACGTCAC	MH96	3’ region *rz* fw	FRT
MS85	GCGGCGGAGCTCCCGATTAAGGAAACTTAGT CG	MH96	2.039 bp 3’ region *rz* rev	SacI
MS86	ATGCGTAAAGGCGAAGAGC	pBAD::sfGFP	sfGFP fw	
MS87	GGATCCTTAATGATGATGATGATGATGTTTG TAC	pBAD::sfGFP	sfGFP rev	Bam HI
MS88	CCCTCTAGAGATTAGATTATGTTCGTTAGCTC	MH96	Hol 5 fw	XbaI
MS89	**CAGCTCTTCGCCTTTACGCAT**CCCCAAGAAAC GCCGCCACAG	MH96	Hol 5 rev	FRT
MS90	**AAACATCATCATCATCATCATTAA**GGATCCAG TCCGGAGAAAAATCGTGGG	MH96	Hol 3 fw	FRT Bam HI
MS91	AAACCCGGGCGTTGCTGCAACCGGATAAC	MH96	Hol 3 rev	XmaI

MS92	AAATCTAGAGGTTCAACAACACGATTCCC	MH96	Rz1 5 fw	XbaI
MS93	**CAGCTCTTCGCCTTTACGCAT**TCCTTTTTGGGGAGACATTATT	MH96	Rz1 5 rev	FRT
MS94	**CAAACATCATCATCATCATCATTAA**GGATCCATCTCTCTTTTAGTCAATTCAG	MH96	Rz1 3 fw	FRT Bam HI
MS95	AAACCCGGGCCTGAACGCAGTAGGAATC	MH96	Rz1 3 rev	XmaI
MS96	AAAGGATCCATGCCCGTTCCATACAGAAGC	pHP45	Spec – BamHI fw	Bam HI
MS97	AAAGGATCCACATTATTTGCCGACTACCT	pHP45	Spec – BamHI rev	Bam HI
MS98	CTGCGTCATTCTGACGTTAAC	MH96	Validation HolA-sfGFP fw	
MS99	GAGAGATTTATCCTTTTTGG	MH96	Validation HolA-sfGFP rev	
AY_F	**TCCATAAGATTAGCGGATCCTAC**	pAY-2	Sequencing primer fw for pAY-2 constructs	
AY_R	**CATGGGGTCAGGTGGCAC**		Sequencing primer rev for pAY-2 constructs	

Bold letters indicate FRT site, underlined indicate restriction sites

References

1. Lorenz C, Gesell T, Zimmermann B, Schoeberl U, Bilusic I, Rajkowitsch L, Waldsich C, von Haeseler A, Schroeder R. 2010. Genomic SELEX for Hfq-binding RNAs identifies genomic aptamers predominantly in antisense transcripts. Nucleic Acids Res 38:3794-3808.
2. Lang B, Blot N, Bouffartigues E, Buckle M, Geertz M, Gualerzi CO, Mavathur R, Muskhelishvili G, Pon CL, Rimsky S, Stella S, Babu MM, Travers A. 2007. High-affinity DNA binding sites for H-NS provide a molecular basis for selective silencing within proteobacterial genomes. Nucleic Acids Res 35:6330-6337.
3. Thoma S, Schobert M. 2009. An improved Escherichia coli donor strain for diparental mating. FEMS Microbiol Lett 294:127-132.
4. Lorow D, Jessee J. 1990. Max efficiency DH10B: a host for cloning methylated DNA. Focus 12:19.
5. Marinus MG, Carraway M, Frey AZ, Brown L, Arraj JA. 1983. Insertion mutations in the *dam* gene of *Escherichia coli* K-12. Mol Gen Genet 192:288-289.
6. Hurst MR, Becher SA, Young SD, Nelson TL, Glare TR. 2011. *Yersinia entomophaga* sp. nov., isolated from the New Zealand grass grub *Costelytra zealandica*. Int J Syst Evol Microbiol 61:844-849.
7. Schoof M, O'Callaghan M, Sheen CR, Glare TR, Hurst MRH. 2022. Identification of genes involved in exoprotein release using a high-throughput exoproteome screening assay in *Yersinia entomophaga*. PLoS One 17:e0263019.
8. Datsenko KA, Wanner BL. 2000. One-step inactivation of chromosomal genes in *Escherichia coli* K-12 using PCR products. Proc Natl Acad Sci U S A 97:6640-6645.
9. Penfold RJ, Pemberton JM. 1992. An improved suicide vector for construction of chromosomal insertion mutations in bacteria. Gene 118:145-146.
10. Shaw RJ, McNeill MM, Maass DR, Hein WR, Barber TK, Wheeler M, Morris CA, Shoemaker CB. 2003. Identification and characterisation of an aspartyl protease inhibitor homologue as a major allergen of *Trichostrongylus colubriformis.* Int J Parasitol 33:1233-1243.
11. Chang AC, Cohen SN. 1978. Construction and characterization of amplifiable multicopy DNA cloning vehicles derived from the P15A cryptic miniplasmid. J Bacteriol 134:1141-1156.
12. Prentki P, Krisch HM. 1984. In vitro insertional mutagenesis with a selectable DNA fragment. Gene 29:303-13.
13. Celli J, Deng W, Finlay BB. 2000. Enteropathogenic *Escherichia coli* (EPEC) attachment to epithelial cells: exploiting the host cell cytoskeleton from the outside. Cell Microbiol 2:1-9.
14. Kilic S, White ER, Sagitova DM, Cornish JP, Erill I. 2014. CollecTF: a database of experimentally validated transcription factor-binding sites in Bacteria. Nucleic Acids Res 42:D156-160.
15. Dudek CA, Jahn D. 2022. PRODORIC: state-of-the-art database of prokaryotic gene regulation. Nucleic Acids Res 50:D295-D302.
